# Serum n-6 polyunsaturated fatty acids and risk of atrial fibrillation: the Kuopio Ischaemic Heart Disease Risk Factor Study

**DOI:** 10.1007/s00394-021-02780-0

**Published:** 2021-12-27

**Authors:** Behnam Tajik, Tomi-Pekka Tuomainen, Masoud Isanejad, Jukka T. Salonen, Jyrki K. Virtanen

**Affiliations:** 1 Institute of Public Health and Clinical Nutrition, University of Eastern Finland, Kuopio Campus, PO Box 1627, 70211 Kuopio, Finland; 2 Institute of Life Course and Medical Sciences, University of Liverpool, Liverpool, L7 8TX England; 3 MAS-Metabolic Analytical Services Oy, Helsinki, Finland; 4 The Faculty of Medicine, Department of Public Health, The University of Helsinki, Helsinki, Finland

**Keywords:** Polyunsaturated fatty acids, n-6 PUFA, Arrhythmia, Atrial fibrillation, Population study

## Abstract

**Purpose:**

N-6 polyunsaturated fatty acids (PUFA), particularly linoleic acid (LA), have been associated with lower risk of coronary heart disease (CHD), but little is known about their antiarrhythmic properties. We investigated the association of the serum n-6 PUFAs with the risk of atrial fibrillation (AF), the most common type of cardiac arrhythmia.

**Methods:**

The study included 2450 men from the Kuopio Ischaemic Heart Disease Risk Factor Study, aged 42–60 years at baseline. The total n-6 PUFA includes linoleic acid (LA), arachidonic acid (AA), γ-linolenic acid (GLA) and dihomo-γ-linolenic acid (DGLA). Cox proportional hazards regression was used to estimate hazard ratio (HR) of incident events.

**Results:**

During the mean follow-up of 22.4 years, 486 AF cases occurred. The multivariable-adjusted HR in the highest versus the lowest quartile of total serum n-6 PUFA concentration was 0.79 (95% CI 0.58–1.08, *P* trend = 0.04). When evaluated individually, only serum LA concentration was inversely associated with AF risk (multivariable-adjusted extreme-quartile HR 0.69, 95% CI 0.51–0.94, *P* trend = 0.02). These associations were stronger among the men without history of CHD or congestive heart failure at baseline, compared to men with such disease history (*P* for interaction = 0.05 for total n-6 PUFA and LA). Similar associations were observed with dietary LA and AA intakes. No significant associations were observed with serum AA, GLA or DGLA concentrations.

**Conclusions:**

Higher circulating concentration and dietary intake of n-6 PUFA, mainly LA, are associated with lower risk of AF, especially among men without history of CHD or congestive heart failure.

**Supplementary Information:**

The online version contains supplementary material available at 10.1007/s00394-021-02780-0.

## Introduction

Atrial fibrillation (AF) is the most common of all sustained cardiac arrhythmias, especially in elderly [1]. AF is associated with fatigue, reduced exercise tolerance, and increased risk of cardiovascular disease (CVD), including congestive heart failure and stroke, as well as total and CVD mortality [2].

The n-6 polyunsaturated fatty acids (PUFA), especially linoleic acid (LA, 18:2n-6), have been shown to be beneficial regarding cardiovascular health [3, 4]. LA, the predominant n-6 PUFA, is an essential fatty acid and it is found mainly in vegetable oils, nuts, and oily seeds [5]. LA can be endogenously converted to γ-linolenic acid (GLA, 18:3n-6), dihomo-γ-linolenic acid (DGLA; 20:3n-6) and arachidonic acid (AA, 20:4n-6) by stepwise desaturation and chain elongation. These fatty acids, except for DGLA, are also obtained from vegetable oils and animal products such as poultry, meat, fish, seafood, and eggs [5, 6].

It has been indicated that higher intake of LA is associated with lower risk of CVD [4, 7, 8]. Knowledge regarding the cardiovascular effects of AA, GLA and DGLA are still less established. However, few studies have investigated the impact of the n-6 PUFA on the risk of AF, with inconsistent results. One prospective cohort study found an inverse association between plasma AA concentrations and risk of AF [9], whereas a Mendelian randomization study found a suggestive inverse association between genetically-predicted LA concentrations and risk of AF. In contrast, in a prospective cohort study dietary LA or AA intakes were not associated with the risk of AF [11], but higher adipose tissue n-6 PUFA concentration (sum of GLA, DGLA and AA) was associated with lower risk of developing AF [12]. However, LA was associated with an increased risk of AF in men, but not in women [12].

Therefore, because of the limited and inconsistent findings, we investigated the prospective associations of the serum n-6 PUFA concentrations and dietary n-6 PUFA intakes with the risk of AF among middle-aged and older men from the population-based KIHD cohort. We have previously shown in the KIHD cohort that higher n-6 PUFA intake or serum concentrations, especially LA, were associated with lower risk of fatal myocardial infarction, CVD death and total mortality [13, 14]. In addition, as presence of CHD and congestive heart failure is strongly associated with the new onset of AF [15], we investigated and compared the associations of the n-6 PUFAs with AF among participants with and without history of coronary heart disease (CHD) and congestive heart failure at the baseline.

## Methods

### Study population

The KIHD is a population-based study designed to investigate risk factors for CVD, atherosclerosis, and related outcomes in men from eastern Finland [16], a total of 2682 men (82.9% of those eligible) who were 42, 48, 54 or 60 years old and living in the city of Kuopio or its surrounding areas participated in the baseline examinations in 1984–1989. The baseline characteristics of the entire study population have been described previously [17]. The KIHD protocol was approved by the Research Ethics Committee of the University of Kuopio and complies with Declaration of Helsinki (ClinicalTrials.gov Identifier: NCT03221127). All the subjects signed a written informed consent. Study participants were not involved in the design, or conduct, or reporting, or dissemination plans of the current study. The manuscript complies with the STROBE checklist. Subjects with a history of AF at baseline (*n* = 32) were excluded from the analyses. We also excluded men with missing data on serum n-6 PUFAs (*n* = 200), leaving 2450 men for the analyses. The stratified analyses based on disease history included 1795 men without history of CHD or congestive heart failure and 655 men with history of these diseases. For the dietary analyses we excluded men with missing data on dietary intakes (*n* = 72), leaving 2610 men for the analyses, 1902 men without history of CHD or congestive heart failure and 708 men with history of these diseases.

### Measurements

Subjects gave venous blood samples between 8 A.M. and 10 A.M. at the baseline examinations. The subjects were instructed to abstain from ingesting alcohol for 3 days and from smoking and eating for 12 h before giving the sample. Comprehensive description of the determination of serum lipids and lipoproteins, assessment of medical history and medications, smoking, and alcohol consumption have been reported previously [18].

Physical activity was evaluated based on the 12-month leisure-time physical activity questionnaire and expressed as kcal/day [19]. The most common leisure-time physical activities were recorded, including the average duration, intensity, and frequency of each activity. BMI was computed as the ratio of weight in kilograms to the square of height in meters. Education and annual income were assessed using self-administered questionnaires. Hypertension diagnosis was defined as systolic/diastolic blood pressure > 140/90 mmHg at study visit or use of hypertension medication [18]. Dietary intakes were assessed using 4-day food recording at the time of blood sampling [13]. Nutrient intakes were estimated using the NUTRICA® 2.5 software (Social Insurance Institution, Turku, Finland). The databank of the software is mainly based on Finnish values of nutrient composition of foods. For the total n-6 PUFA intake, we used the sum of LA and AA intakes. The database does not have information on GLA or DGLA content in foods. Healthy Nordic diet score used in the analyses is based on the Baltic Sea diet score [20] but modified slightly to comply with the availability of the dietary data in the KIHD cohort [21]. The score ranges from 0 to 25. The higher the score, the higher the adherence to a healthy Nordic diet.

### Serum fatty acid measurements

Serum esterified and nonesterified fatty acids were measured in 1991 from samples that had been stored at − 80 °C in one gas chromatographic run without preseparation, as described previously [22]. Serum fatty acids were extracted with chloroform–methanol. Chloroform phase was evaporated and treated with sodium methoxide, which methylated esterified fatty acids. Quantification was carried out with reference standards (Check Prep Inc., Elysian, MN). Each analyte had individual reference standard, and an internal standard was eicosan. Fatty acids were chromatographed in an NB-351 capillary column (HNU-Nordion, Helsinki, Finland) by a Hewlett-Packard 5890 Series II gas chromatograph (Hewlett-Packard Company, Avondale, PA, since 1999 Agilent Technologies Inc.) with a flame ionization detector. Results were obtained in micromoles per litter and in the data analyses proportion of the fatty acid of the total serum fatty acids was used. The interassay coefficient of variation (CVs) for repeated measurements were 8.7% for LA, 11.6% for GLA, 8.3% for DGLA, and 9.9% for AA. For the serum total n-6 PUFA concentration, we used the sum of LA, GLA, DGLA and AA.

### Ascertainment of follow-up events

All AF events that occurred between study entry and December 31, 2014, were included. Data on events were obtained by record linkage from the national computerized hospitalization registry, which covers every hospitalization in Finland. Subjects were hospitalized because of AF or had AF when they were hospitalized for other reasons. Data on vital status were obtained from Statistics Finland. Cardiovascular causes of AF were coded according to International Classification of Diseases codes (8th revision code 427.4, 9th revision code 427.3, and 10th revision code I48) and the accuracy was verified by a physician [23].

### Statistical analysis

The univariate associations of the serum n-6 PUFA with demographic, lifestyle and clinical characteristics at baseline were assessed by means and linear regression for continuous variables and Chi^2^-test for categorical variables. Correlations between the individual n-6 PUFAs were evaluated by Spearman correlation. Cox proportional hazards regression models adjusted for relevant covariates were used to estimate hazard ratios (HRs) of incident events. The validity of the proportional hazard assumption was evaluated using Schoenfeld residuals, and the assumptions were met. The analyses were controlled for possible confounders, which were selected based on established risk factors for AF [15], or on associations with exposures or outcomes in the present analysis.

Two different models were used to control for confounding factors. The model 1 was adjusted for age (years) and examination year. The multivariable model 2 included model 1 and BMI (kg/m^2^), smoking (pack/years), years of education, leisure-time physical activity (kilocalories/day), intake of alcohol (grams/week), serum triglycerides (mmol/L), serum long-chain n-3 PUFA concentration (% of all serum fatty acids), systolic and diastolic blood pressure (mm Hg), family history of ischemic heart disease, and use of hypercholesterolemia or hypertension medications at baseline or during follow-up (yes or no). In the analyses with dietary n-6 PUFA intakes, also total energy intake was used as a covariate. Additional adjustments for serum HDL cholesterol or LDL cholesterol concentrations, income, history of type 2 diabetes, or intakes of fruits, berries and vegetables, eggs, whole grains, meat, fish, dairy, butter, or a healthy Nordic diet score did not appreciably change the associations (< 5% change in estimates).”

Cohort means were used to replace missing values in covariates (< 0.5%). Statistical significance of the interactions on a multiplicative scale was assessed by likelihood ratio tests with a cross-product term. Tests of linear trend across categories were conducted by assigning the median values for each category of exposure variable and treating those as a single continuous variable. Potential nonlinear associations were assessed semi-parametrically using restricted cubic splines. All *P* values were two-sided (*α* = 0.05). Data were analyzed using the SPSS software version 27 for windows (Armonk, NY: IBM Corp.) and Stata version 14.1 for spline analysis (Stata Corp).

## Results

### Baseline characteristics

The mean (SD) age of the participants was 53.0 (5.2) years. The mean (SD) serum concentrations, as a percentage of all serum fatty acids, were 32.79% (4.85) for serum total n-6 PUFA concentration, 26.40% (4.65) for LA, 0.29% (0.11) for GLA, 1.34% (0.30) for DGLA, and 4.77% (1.01) for AA. The intercorrelations between the individual n-6 PUFA were weak, except for a moderate correlation between GLA and DGLA; (−0.19 for LA and GLA, *P* < 0.001), (− 0.09 for LA and DGLA, *P* < 0.001), (0.13 for LA and AA, *P* < 0.001), (0.54 for GLA and DGLA, *P* < 0.001), (0.19 for GLA and AA, *P* < 0.001), (0.09 for DGLA and AA, *P* < 0.001).

Baseline characteristics of the participants according to quartiles of the total n-6 PUFA concentration are presented in the Table 1. Men with higher concentration were more likely to have a higher annual income and education, leisure-time physical activity, and serum HDL and LDL cholesterol concentrations, higher intakes of LA and AA, fruits, berries and vegetables, eggs, total grains, whole grains, red meat and total meat products, and a higher adherence to a healthy Nordic diet, but lower serum triglyceride concentration and systolic and diastolic blood pressure, and lower intakes of fish, dairy products, and butter. They also had a lower BMI and alcohol intake and were less likely to have hypertension, congestive heart failure, ischemic heart disease, diabetes, and to smoke.

**Table 1:** Baseline characteristics according to serum total n-6 polyunsaturated fatty acids

Variables	Serum total n-6 polyunsaturated fatty acids quartile (%)				*P* trend
	1 (< 27.3)	2 (27.3–31.5)	3 (31.5–34.4)	4 (> 34.4)	
Number of the subjects	612	613	613	612	
Biochemical variables and other risk factors					
Age (y)	53.6 (4.8)	53.2 (5.1)	52.9 (5.1)	52.3 (5.6)	< 0.001
Education (years)	8.4 (3.1)	8.2 (3.1)	8.8 (3.6)	9.2 (3.7)	< 0.001
Income (euro)	13,029 (8120)	14,235 (9441)	14,744 (9511)	15,440 (10,229)	< 0.001
BMI (kg/m^2^)	28.5 (4.1)	27.2 (3.4)	26.3 (3.1)	25.7 (2.9)	< 0.001
Physical activity (kcal/day)	132.3 (179.1)	129.6 (155.4)	137.3 (169.8)	163.7 (192.9)	0.001
Smoking (%)	34.2%	31.1%	32.2%	28.3%	< 0.001
Serum triglyceride concentration (mmol/l)	1.99 (1.2)	1.29 (0.56)	1.08 (0.46)	0.93 (0.39)	< 0.001
Serum HDL cholesterol (mmol/l)	1.20 (0.31)	1.28 (0.27)	1.33 (0.29)	1.37 (0.30)	< 0.001
Serum LDL cholesterol (mmol/l)	3.90 (1.01)	4.08 (1.01)	4.09 (0.97)	4.07 (1.04)	0.002
Mean systolic blood pressure (mm Hg)	138 (17)	135 (17)	133 (17)	132 (17)	< 0.001
Mean diastolic blood pressure (mm Hg)	91 (11)	89 (10)	88 (10)	87 (10)	< 0.001
Serum LA (% of all serum fatty acids)	20.68 (2.62)	24.98 (1.41)	27.87 (1.35)	32.06 (2.65)	< 0.001
Serum GLA (% of all serum fatty acids)	0.31 (0.12)	0.29 (0.10)	0.27 (0.10)	0.28 (0.11)	< 0.001
Serum DGLA (% of all serum fatty acids)	1.32 (0.26)	1.36 (0.37)	1.35 (0.28)	1.32 (0.27)	0.95
Serum AA (% of all serum fatty acids)	4.26 (0.98)	4.79 (0.96)	4.93 (0.94)	5.10 (0.97)	< 0.001
Serum long-chain n-3 PUFA (%)	4.7 (1.9)	4.8 (1.7)	4.8 (1.5)	4.4 (1.2)	0.007
Dietary factors^a^					
LA (% of total energy intake)	2.83 (1.20)	3.04 (1.24)	3.31 (1.22)	3.96 (1.29)	< 0.001
AA (% of total energy intake)	0.06 (0.30)	0.07 (0.31)	0.07 (0.32)	0.08 (0.33)	< 0.001
Fish (g/d)	52 (67)	46 (50)	46 (52)	41 (47)	0.001
Fruits, berries and vegetables (g/d)	226 (149)	251 (162)	258 (160)	274 (154)	< 0.001
Dairy (g/d)	750 (390)	722 (371)	706 (338)	652 (340)	< 0.001
Eggs (g/d)	29 (26)	30 (24)	35 (26)	33 (25)	0.002
Total grains (g/d)	237 (92)	249 (97)	255 (85)	273 (96)	0.001
Whole grains (g/d)	152 (75)	155 (80)	159 (70)	171 (79)	< 0.001
Meat^b^ (g/d)	148 (73)	151 (73)	157 (82)	161 (86)	0.002
Red meat (g/d)	140 (74)	142 (71)	147 (79)	148 (83)	0.002
Butter (g/d)	37 (27)	36 (29)	33 (25)	24 (25)	< 0.001
Healthy Nordic diet score^b^	12	12	13	13	< 0.001
Alcohol intake (g/w)	96.9 (149.5)	76.5 (122.9)	69.1 (147.3)	53.4 (85.1)	< 0.001
Diseases					
Coronary heart disease (%)	31.7%	24.0%	20.4%	21.9%	< 0.001
Congestive heart failure (%)	12.1%	5.9%	6.4%	3.6%	< 0.001
Diabetes (%)	10.3%	5.5%	3.9%	3.4%	< 0.001
Treated hypertension (%)	72.9%	62.6%	55.3%	52.0%	< 0.001

Values are means (SD) or percentages

*AA* arachidonic acid, *DGLA* dihomo-γ-linolenic acid, *GLA* γ-linolenic acid, *HDL* high density lipoprotein, *LA* linoleic acid, *LDL* low density lipoprotein, *PUFA* polyunsaturated fatty acids

^a^4-day mean

^b^Includes processed and unprocessed red and white meat and game

^c^The score ranges from 0 to 25. The higher the score, the higher the adherence to a healthy Nordic diet

### Associations between serum the n-6 PUFA and risk of AF

**Fig. 1:**
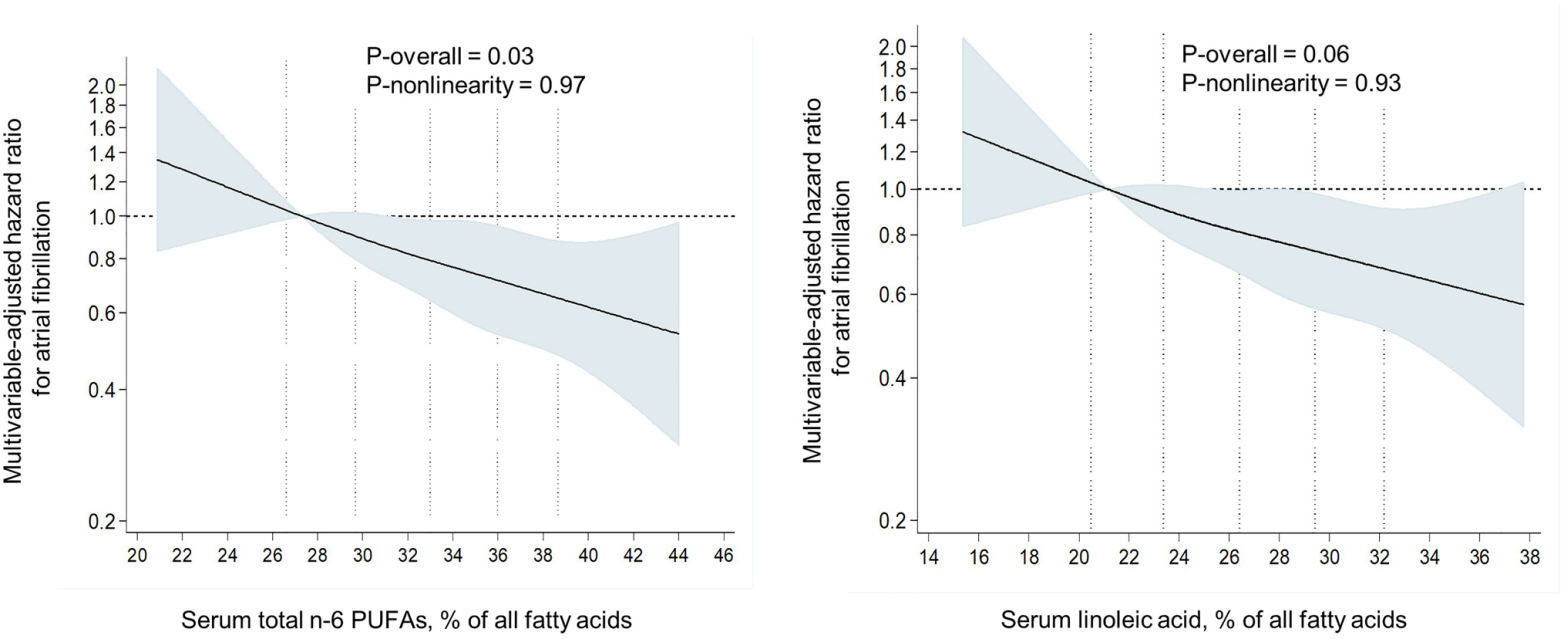
Multivariable-adjusted hazard ratios of serum n–6 PUFAs and serum LA with risk of atrial fibrillation among 2450 men, evaluated by restricted cubic splines from Cox proportional hazards models. The models were adjusted for age (years), examination year, body mass index (kg/m^2^), smoking (pack/years), years of education, leisure-time physical activity (kilocalories/day), intake of alcohol (grams/week), serum triglycerides (mmol/L), serum long-chain n-3 PUFA concentration (%), systolic and diastolic blood pressures (mm Hg), family history of ischemic heart disease, and use of hypercholesterolemia or hypertension medications at baseline or during follow-up (yes or no). The solid lines represent the central risk estimates, and the shaded areas represent 95% CIs, relative to the reference level (12.5th percentile). The dotted vertical lines correspond to the 10th, 25th, 50th, 75th and 90th percentiles of fatty acid concentrations. Abbreviations: LA, linoleic acid; PUFA, polyunsaturated fatty acids

**Fig. 2:**
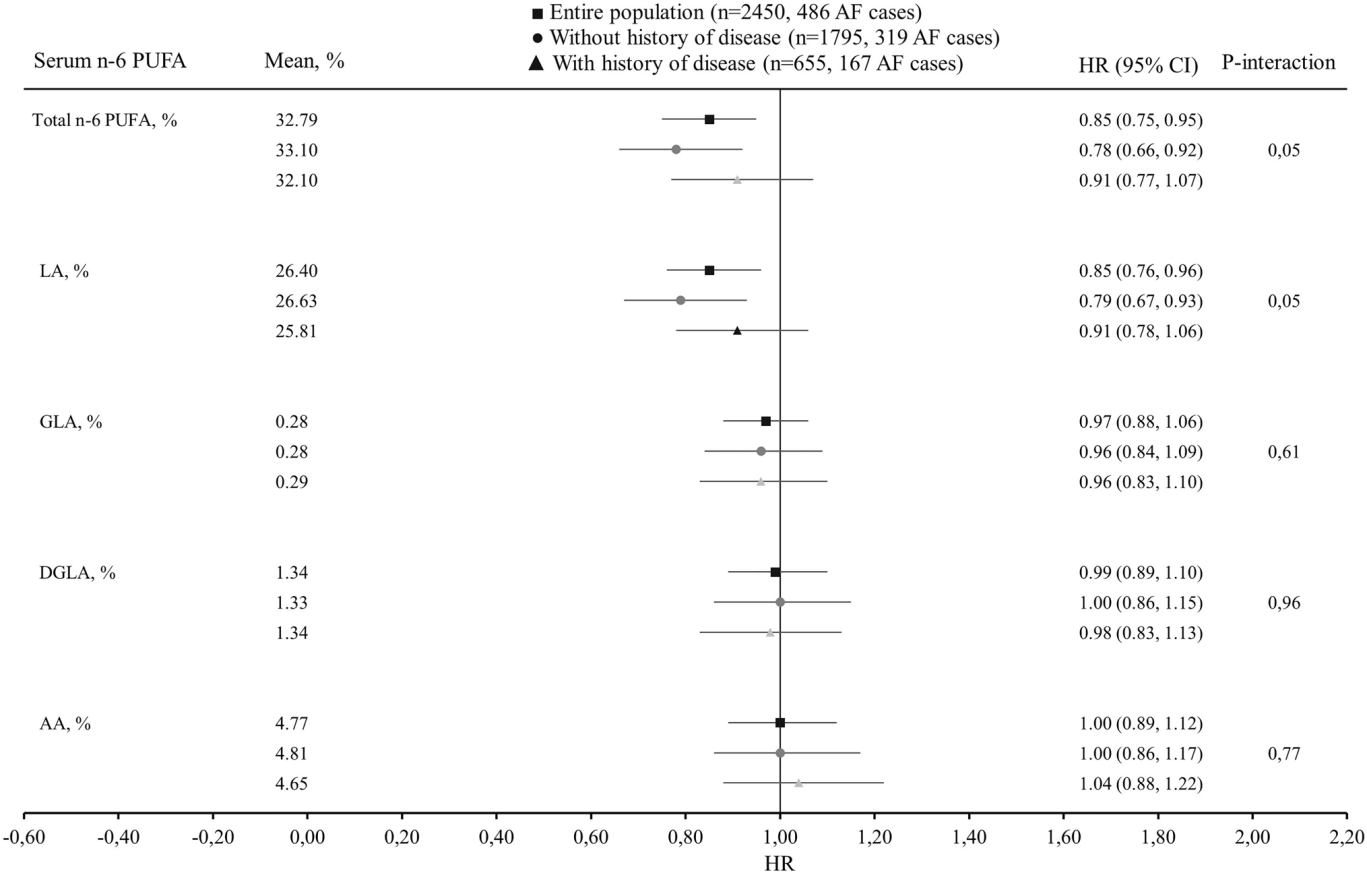
Multivariable-adjusted hazard ratios of atrial fibrillation per 1 SD increase in the serum fatty acid concentrations. The models were adjusted for age (years), examination year, body mass index (kg/m^2^), smoking (pack/years), years of education, leisure-time physical activity (kilocalories/day), intake of alcohol (grams/week), serum triglycerides (mmol/L), serum long-chain n-3 PUFA concentration (%), systolic and diastolic blood pressures (mm Hg), family history of ischemic heart disease, and use of hypercholesterolemia or hypertension medications at baseline or during follow-up (yes or no). Abbreviations: AA, arachidonic acid; DGLA, dihomo-γ-linolenic acid; GLA, γ-linolenic acid; LA, linoleic acid; PUFA, polyunsaturated fatty acids

During the mean follow-up of 22.4 years, AF was diagnosed in 486 (19.8%) of the 2450 men. The risk for AF was 33% lower (HR 0.76, 95% CI 0.59–0.99, *P* trend across quartiles = 0.007) in the highest vs. the lowest serum total n-6 PUFA quartile after adjustment for age and examination year (Model 1, Table 2). Further adjustments for potential confounders slightly attenuated the association (extreme-quartile HR 0.79, 95% CI 0.58–1.08, *P* trend = 0.04) (Model 2, Table 2). Among the individual fatty acids, only LA was associated with reduced risk of AF (the multivariable-adjusted extreme-quartile HR 0.69, 95% CI 0.51–0.94, *P* trend = 0.02) (Model 2, Table 2). GLA, DGLA and AA were not associated with the risk of AF (Table 2). Similarly, when evaluated continuously, 1-SD increase in serum total n-6 PUFA and serum LA concentrations were associated with 15% lower risk of AF (HR 0.85, 95% CI 0.75–0.95 and HR 0.85, 95% CI 0.76–0.96, respectively) (Fig. 2). Restricted cubic splines analysis confirmed a relatively linear inverse association of serum total n-6 PUFA and LA with the risk of AF and showed little evidence for nonlinearity (Fig. 1). No associations were observed between GLA, DGLA, AA and risk of AF (Table 2, Supplemental Fig. 1).

**Table 2:** Risk of atrial fibrillation in quartiles of serum n-6 polyunsaturated fatty acids among 2450 men from the Kuopio Ischaemic Heart Disease Risk Factor Study

	Quartile				*P* trend
	1 (*n* = 549)	2 (*n* = 550)	3 (*n* = 550)	4 (*n* = 550)	
Total n-6 PUFA, %	< 29.66	29.66–33.01	33.01–35.98	> 35.98	
Number of events	123	143	107	113	
Model 1	1 (reference group)	1.02 (0.81–1.31)	0.73 (0.56–0.94)	0.76 (0.59–0.99)	0.01
Model 2	1 (reference group)	1.05 (0.81–1.36)	0.75 (0.56–1.00)	0.79 (0.58–1.08)	0.04
LA, %	< 24.04	24.04–26.42	26.42–28.77	> 28.77	
Number of events	128	130	125	103	
Model 1	1 (reference group)	0.86 (0.68–1.10)	0.85 (0.66–1.09)	0.66 (0.51–0.85)	0.002
Model 2	1 (reference group)	0.89 (0.69–1.16)	0.89 (0.67–1.17)	0.69 (0.51–0.94)	0.02
GLA, %	< 0.22	0.22–0.27	0.27–0.33	> 0.33	
Number of events	117	129	125	115	
Model 1	1 (reference group)	1.10 (0.86–1.42)	1.13 (0.88–1.45)	1.05 (0.81–1.36)	0.75
Model 2	1 (reference group)	1.08 (0.84–1.38)	1.10 (0.85–1.41)	1.05 (0.81–1.36)	0.77
DGLA, %	< 1.19	1.19–1.32	1.32–1.46	> 1.46	
Number of events	109	129	127	121	
Model 1	1 (reference group)	1.27 (0.98–1.63)	1.12 (0.86–1.44)	1.16 (0.90–1.50)	0.43
Model 2	1 (reference group)	1.27 (0.98–1.64)	1.07 (0.82–1.39)	1.11 (0.85–1.45)	0.74
AA, %	< 4.22	4.22–4.73	4.73–5.27	> 5.27	
Number of events	120	133	111	122	
Model 1	1 (reference group)	1.08 (0.84–1.38)	0.86 (0.66–1.11)	0.97 (0.75–1.25)	0.48
Model 2	1 (reference group)	1.11 (0.87–1.43)	0.88 (0.67–1.14)	0.98 (0.74–1.29)	0.51

Values are hazard ratios (95% confidence interval)

Model 1: adjusted for age and examination year

Model 2: adjusted for model 1 plus body mass index, smoking, leisure-time physical activity, education, alcohol intake, serum triglycerides, systolic and diastolic blood pressures, serum long-chain n-3 polyunsaturated fatty acids, family history of ischemic heart disease, and use of hypercholesterolemia or hypertension medications

### Sensitivity analyses

The associations of the total n-6 PUFA and LA were slightly stronger among the subjects without history of CHD or congestive heart failure at baseline, whereas no statistically significant associations were observed among the men with history of these diseases (*P*-interaction = 0.05, Fig. 2). Among the 1795 men without disease history, 319 AF events occurred. Each 1-SD increase in serum total n-6 PUFA and LA concentrations were associated with a multivariable-adjusted 22% (95% CI 8–34%) and 21% (95% CI 7–33%) lower risk of AF, respectively (Fig. 2). There were no statistically significant associations between the serum GLA, DGLA and AA and risk of AF in these analyses, either (*P* for interactions ≥ 0.61) (Fig. 2).

### Associations between dietary n-6 PUFA intakes and risk of AF

During the follow-up, among the 1902 men without disease history, 335 AF events occurred, while AF was diagnosed in 176 of the 708 men with disease history. Similar associations with the risk of AF as with the serum n-6 PUFA concentrations were also observed with the intakes of total n-6 PUFA, LA and AA. Higher intakes of total n-6 PUFA and LA were associated with reduced risk of AF [the multivariable-adjusted extreme-quartile HR = 0.66 (95% CI 0.43–0.98, *P* trend = 0.04) for total n-6 PUFA, and HR = 0.64 (95% CI 0.43–0.95, *P* trend = 0.03)] for LA, only among those without history of CHD or congestive heart failure at baseline (Supplemental Table 1). There were no statistically significant associations between the dietary intake of AA and risk of AF among men with or without disease history (Supplemental Table 1).

## Discussion

In this population-based cohort study among middle-aged and older men from eastern Finland, higher serum concentration and dietary intake of LA, the major n-6 PUFA, were associated with lower risk of AF and the associations were evident especially among those without history of CHD and congestive heart failure. Serum GLA, DGLA, and AA were not associated with the risk.

Only a few studies have evaluated the associations of the n-6 PUFA with the risk of AF, with heterogeneous findings. In a multi-ethnic cohort of 6229 participants free of CVD (813 AF cases), plasma concentrations of n-6 PUFAs, particularly AA, were associated with lower risk of incident AF [9]. In a Mendelian randomization study among 588,190 individuals (65,446 AF cases), genetically-predicted higher plasma LA concentration, but not AA concentration, had a suggestive inverse association with the risk of AF [10]. This finding with LA is in contrast with the results of the Diet, Cancer and Health cohort study among 57,053 Danish individuals aged 50–65 years (2274 AF cases), where intakes of LA and AA were not associated with the risk of AF [11]. However, in another study from this Danish cohort, adipose tissue concentration of the minor n-6 PUFAs (sum of GLA, DGLA and AA) was associated with a lower risk of developing AF (4710 AF cases) [12]. In contrast, LA was associated with an increased risk of AF in men, but not in women [12].

Although mechanisms underlying the possible antiarrhythmic properties of LA are not well established and future research is needed, some potential factors to explain the inverse association between serum LA and risk of AF could include the favorable impact of LA on the lipoprotein metabolism [24], atrial ischemia [25], and blood pressure [26] that have been shown to be associated with the risk of AF [27–29]. Other potential mechanisms include the beneficial properties of LA in the reduction of arterial stiffness and vascular resistance, promotion of endothelium-vasodilation and improvement of pulse wave velocity, which are related to the risk of cardiac arrhythmias, especially among healthy populations [30, 31].

Presence of CHD and congestive heart failure are identified to be independent predictors of AF development [15]. In the present study, the inverse association between serum total n-6 PUFA and LA and risk of AF was observed mainly among those without history of CHD and congestive heart failure. This might be partially explained by the favorable systematic changes in the modifiable risk factors, e.g., diet modification, of AF in participants with the history of cardiac events, which may weaken the association of total n-6 PUFA and LA with the risk of AF. Moreover, this finding suggests that the observed inverse association between serum LA and risk of AF is not mediated by the impact of n-6 PUFAs on the risk of prior cardiac events, specifically CHD and congestive heart failure.

A strength of this study is the use of serum n-6 PUFA in addition to dietary intakes, which also enabled us to investigate the associations with GLA and DGLA. Serum fatty acids are objective biomarkers for exposure and may thus reduce the bias by misclassification that would reduce the associations towards the null in the analyses with dietary intakes. However, analyses with both the serum concentrations and the dietary intakes gave congruent results. Other strengths include the population-based recruitment, extensive examination of potential confounders, and relatively large numbers of incident AF events.

A limitation in this study was the single measurement of fatty acids and assessment of dietary intakes at baseline for all men, which may cause random error and attenuate the associations. However, relatively strong correlations (*r* ≥ 0.5) for all n-6 PUFAs were previously observed among the subgroup of men with repeated measurements after 4 and 11 years during the follow-up in KIHD [14]. The participants were middle-aged and older men from Eastern Finland, so the findings may not be generalizable to other populations or to women. Also, because of the observational study design, conclusions about causality cannot be drawn.

In conclusion, higher serum LA concentration and higher dietary LA intake were associated with a lower risk of AF, a common cardiac arrhythmia. The association was more evident among the men without history of CHD or congestive heart failure. AA and the minor n–6 PUFAs, GLA and DGLA, had no association with AF risk. More large-scale studies in diverse populations are needed to confirm these findings and explore the underlying mechanisms.

## Supplementary Information

Below is the link to the electronic supplementary material.

Supplementary figure 1. (JPG 324 KB). Multivariable-adjusted hazard ratios of serum AA, DGLA, and GLA with risk of atrial fibrillation among 2450 men, evaluated by restricted cubic splines from Cox proportional hazards models. The models were adjusted for age (years), examination year, body mass index (kg/m^2^), smoking (pack/years), years of education, leisure-time physical activity (kilocalories/day), intake of alcohol (grams/week), serum triglycerides (mmol/L), serum long-chain n-3 PUFA concentration (%), systolic and diastolic blood pressures (mm Hg), family history of ischemic heart disease, and use of hypercholesterolemia or hypertension medications at baseline or during follow-up (yes or no). The solid lines represent the central risk estimates, and the shaded areas represent 95% CIs, relative to the reference level (12.5th percentile). The dotted vertical lines correspond to the 10th, 25th, 50th, 75th and 90th percentiles of fatty acid concentrations. Abbreviations: AA, arachidonic acid; DGLA, dihomo-γ-linolenic acid; GLA, γ -linolenic acid.

Supplementary file2 (DOCX 26 KB)

## Abbreviations

AA: Arachidonic acid
DGLA: Dihomo-γ-linolenic acid
GLA: γ-Linolenic acid
LA: Linoleic acid
KIHD: Kuopio Ischaemic Heart Disease Risk Factor Study
PUFA: Polyunsaturated fatty acids
