# Supplementary figures and images for: Serum n-6 polyunsaturated fatty acids and risk of atrial fibrillation: the Kuopio Ischaemic Heart Disease Risk Factor Study
Source: Eur J Nutr. 2021 Dec 27;61(4):1981–9. doi: 10.1007/s00394-021-02780-0 (PMC9106603; doi:10.1007/s00394-021-02780-0)

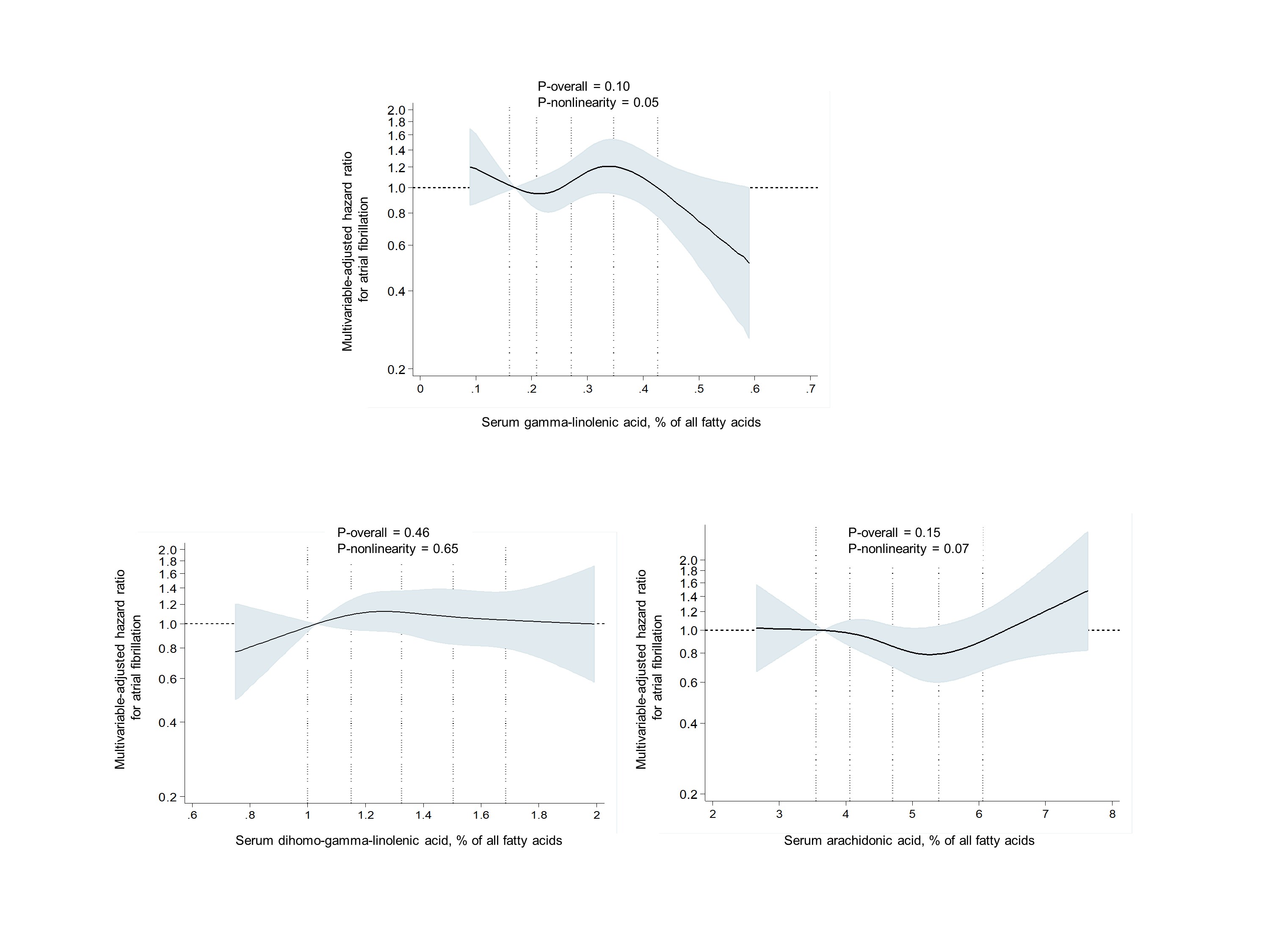

Supplement: Supplementary file 1 — Supplementary figure 1. (JPG 324 KB). Multivariable-adjusted hazard ratios of serum AA, DGLA, and GLA with risk of atrial fibrillation among 2450 men, evaluated by restricted cubic splines from Cox proportional hazards models. The models were adjusted for age (years), examination year, body mass index (kg/m2), smoking (pack/years), years of education, leisure-time physical activity (kilocalories/day), intake of alcohol (grams/week), serum triglycerides (mmol/L), serum long-chain n-3 PUFA concentration (%), systolic and diastolic blood pressures (mm Hg), family history of ischemic heart disease, and use of hypercholesterolemia or hypertension medications at baseline or during follow-up (yes or no). The solid lines represent the central risk estimates, and the shaded areas represent 95% CIs, relative to the reference level (12.5th percentile). The dotted vertical lines correspond to the 10th, 25th, 50th, 75th and 90th percentiles of fatty acid concentrations. Abbreviations: AA, arachidonic acid; DGLA, dihomo-γ-linolenic acid; GLA, γ -linolenic acid. [file 394_2021_2780_MOESM1_ESM.jpg]
