# Supplementary material for: Serum n-6 polyunsaturated fatty acids and risk of atrial fibrillation: the Kuopio Ischaemic Heart Disease Risk Factor Study
Source: Eur J Nutr. 2021 Dec 27;61(4):1981–9. doi: 10.1007/s00394-021-02780-0 (PMC9106603; doi:10.1007/s00394-021-02780-0)
Supplement: Supplementary file 2 — Supplementary file2 (DOCX 26 KB) [file 394_2021_2780_MOESM2_ESM.docx]

| **Supplemental Table 1** Multivariable-adjusted hazard ratios of atrial fibrillation in quartiles of n-6 polyunsaturated fatty acid intakes (% of total energy intake)^a^ | | | | | |
| --- | --- | --- | --- | --- | --- |
|  | | n-6 polyunsaturated fatty acid quartile | | | *P* trend |
|  | 1 | 2 | 3 | 4 |  |
| *Without history of CVD* | |  | | |  |
| *Total n-6 PUFA, %* | <2.38 | 2.38-3.17 | 3.17-4.18 | >4.18 |  |
| Number of events (number of subjects) | 92 (475) | 87 (476) | 85 (476) | 71 (475) |  |
| *Hazard ratio (95% CI)* | 1(reference group) | 0.98 (0.69 to 1.39) | 0.87 (0.61 to 1.25) | 0.66 (0.44 to 0.98) | 0.04 |
|  |  |  |  |  |  |
| *With history of CVD* |  |  |  |  |  |
| *Total n-6 PUFA, %* | <2.20 | 2.20-3.09 | 3.09-4.17 | >4.17 |  |
| Number of events (number of subjects) | 42 (177) | 46 (177) | 44 (177) | 44 (177) |  |
| *Hazard ratio (95% CI)* | 1(reference group) | 1.38 (0.95 to 1.99) | 0.93 (0.72 to 1.28) | 1.32 (0.90 to 1.94) | 0.39 |
|  |  |  |  |  |  |
| *Without history of disease* |  |  |  |  |  |
| *LA, %* | <2.32 | 2.32-3.11 | 3.11-4.09 | >4.09 |  |
| Number of events (number of subjects) | 92 (475) | 86 (476) | 86 (476) | 71 (475) |  |
| *Hazard ratio (95% CI)* | 1(reference group) | 0.96 (0.68 to 1.37) | 0.93 (0.65 to 1.33) | 0.64 (0.43 to 0.95) | 0.03 |
|  |  |  |  |  |  |
| *With history of CVD* |  |  |  |  |  |
| *LA, %* | <2.14 | 2.14-3.03 | 3.03-4.08 | >4.08 |  |
| Number of events (number of subjects) | 42 (177) | 45 (177) | 44 (177) | 45 (177) |  |
| *Hazard ratio (95% CI)* | 1(reference group) | 1.38 (0.96 to 1.98) | 0.98 (0.66 to 1.45) | 1.36 (0.91 to 2.00) | 0.27 |
|  |  |  |  |  |  |
| *Without history of disease* |  |  |  |  |  |
| *AA, %* | <0.05 | 0.05-0.06 | 0.06-0.09 | >0.09 |  |
| Number of events (number of subjects) | 88 (475) | 91 (476) | 90 (476) | 66 (475) |  |
| *Hazard ratio (95% CI)* | 1(reference group) | 1.09 (0.77 to 1.53) | 1.03 (0.72 to 1.46) | 0.87 (0.49 to 1.24) | 0.21 |
|  |  |  |  |  |  |
| *With history of disease* |  |  |  |  |  |
| *AA, %* | <0.05 | 0.05-0.06 | 0.06-0.09 | >0.09 |  |
| Number of events (number of subjects) | 44 (177) | 45 (177) | 39 (177) | 48 (177) |  |
| *Hazard ratio (95% CI)* | 1(reference group) | 1.01 (0.70 to 1.44) | 0.73 (0.49 to 1.09) | 1.00 (0.67 to 1.51) | 0.67 |
| ^a^Values are hazard ratios (95% confidence interval).  Model: Adjusted for age, examination year, body mass index, smoking, leisure-time physical activity, education, alcohol intake, serum triglycerides, systolic and diastolic blood pressures, serum long-chain n-3 polyunsaturated fatty acids, total energy intake, family history of ischemic heart disease, and use of hypercholesterolemia or hypertension medications. | | | | | |
